# Near-island biological hotspots in barren ocean basins

**DOI:** 10.1038/ncomms10581

**Published:** 2016-02-16

**Authors:** Jamison M. Gove, Margaret A. McManus, Anna B. Neuheimer, Jeffrey J. Polovina, Jeffrey C. Drazen, Craig R. Smith, Mark A. Merrifield, Alan M. Friedlander, Julia S. Ehses, Charles W. Young, Amanda K. Dillon, Gareth J. Williams

**Affiliations:** 1 Ecosystems and Oceanography Program, Pacific Islands Fisheries Science Center, 1845 Wasp Blvd Building 176, Honolulu, 96818 Hawaii, USA; 2 Department of Oceanography, University of Hawai‘i at Mānoa, 1000 Pope Road, Marine Sciences Building, Honolulu, 96822 Hawaii, USA; 3 Joint Institute for Marine and Atmospheric Research, University of Hawai‘i at Mānoa, 1000 Pope Road, Marine Sciences Building, Honolulu, 96822 Hawaii, USA; 4 Fisheries Ecology Research Laboratory, Department of Biology, University of Hawai‘i at Mānoa, 2538 McCarthy Mall, Honolulu, 96822 Hawaii, USA; 5 Pristine Seas, National Geographic Society, 1145 17th St NW, Washington, DC 20036, USA; 6 Coral Reef Ecosystem Program, Pacific Islands Fisheries Science Center, 1845 Wasp Blvd Building 176, Honolulu, 96818 Hawaii, USA; 7 Center for Marine Biodiversity and Conservation, Scripps Institution of Oceanography, UC San Diego, 9500 Gilman Drive, La Jolla, 92093 California, USA; 8 School of Ocean Sciences, Bangor University, Menai Bridge, LL59 5AB Anglesey, UK

## Abstract

Phytoplankton production drives marine ecosystem trophic-structure and global fisheries yields. Phytoplankton biomass is particularly influential near coral reef islands and atolls that span the oligotrophic tropical oceans. The paradoxical enhancement in phytoplankton near an island-reef ecosystem—Island Mass Effect (IME)—was first documented 60 years ago, yet much remains unknown about the prevalence and drivers of this ecologically important phenomenon. Here we provide the first basin-scale investigation of IME. We show that IME is a near-ubiquitous feature among a majority (91%) of coral reef ecosystems surveyed, creating near-island ‘hotspots' of phytoplankton biomass throughout the upper water column. Variations in IME strength are governed by geomorphic type (atoll vs island), bathymetric slope, reef area and local human impacts (for example, human-derived nutrient input). These ocean oases increase nearshore phytoplankton biomass by up to 86% over oceanic conditions, providing basal energetic resources to higher trophic levels that support subsistence-based human populations.

## Abstract (web summary)

The Island Mass Effect (IME), where island proximity enhances phytoplankton biomass, remains a poorly understood phenomenon. Here, the authors show the IME is a common feature among Pacific reefs, driving increased production that creates biological hotspots in otherwise barren ocean basins.

## Graphical abstract

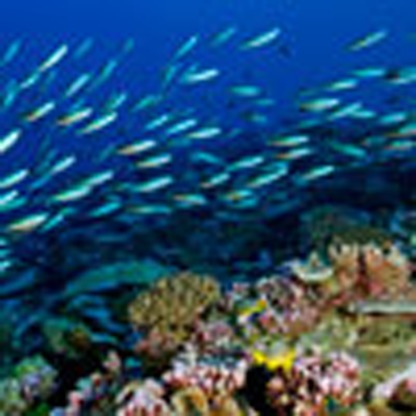

Phytoplankton production is an essential source of energy in the marine environment^1^. The extent and availability of phytoplankton biomass drives the trophic-structure of entire marine ecosystems^2^, dictating the distribution and production of the world's fisheries^3^. The ecological impacts of enhanced phytoplankton biomass are especially acute near tropical coral reef islands and atolls as these ecosystems predominantly reside in nutrient impoverished waters that lack new production^4^. For example, across the central and western Pacific, islands and atolls exposed to elevated levels of nearshore phytoplankton support higher fish biomass and a greater abundance of reef-building organisms than those found in more oligotrophic waters^5^,^6^. Hence, mechanisms that act to promote nearshore phytoplankton biomass are critical for coral reef ecosystem development and persistence^5^.

The increase in phytoplankton biomass proximate to island-reef ecosystems—‘Island Mass Effect' (IME)—was first documented over a half century ago^7^ (Fig. 1). Much of our current knowledge of the IME, however, stems from studies in a small number of locations in geographically confined areas^8^,^9^,^10^. Thus, whether or not the IME is a pervasive phenomenon across broad gradients in oceanic conditions has historically remained unknown. Furthermore, the relative influence of natural vs anthropogenic drivers of variations in the magnitude of the IME has remained a mystery until now.

Here we present a basin-scale investigation of the 60-year-old IME hypothesis. Using 35 coral reef islands and atolls spanning 43° of latitude and 60° of longitude (Fig. 2 and Supplementary Table 1) that cross multiple gradients in oceanic forcing^11^, geophysical attributes^11^, reef-community composition^5^,^12^ and local human impacts^12^, we quantify the prevalence of the IME across the tropical Pacific. We use long-term satellite-derived observations of chlorophyll-*a* (a proxy for phytoplankton biomass) to show the IME is a near-ubiquitous feature and identify key biogeophysical drivers of variations in the magnitude of the IME among Pacific island- and atoll-reef ecosystems. We also incorporate ship-based surveys at 29 of these locations to confirm that the nearshore enhancement in chlorophyll-*a* occurs over the full euphotic depth range. Finally, we show that the IME increases the nearshore standing stock of phytoplankton biomass by up to 85.6% over background oceanic conditions, providing basal energy sources for higher trophic levels across an otherwise barren ocean landscape.

## Results

### Nearshore phytoplankton enhancement

We found that 91% (*n*=32) of island- and atoll-reef ecosystems displayed localized nearshore enhancement in long-term chlorophyll-*a* associated with the IME. The magnitude of the IME varied among locations (evidenced by differences in the linear slope of log–log transformed data, F_1,32_=22.24, *P<*0.0001, Fig. 3a,b, see Methods section). To identify the proximate drivers of the IME, we quantified a suite of biogeophysical predictor variables for each island- and atoll-reef ecosystem (Supplementary Table 2), namely latitude, land area, reef area, bathymetric slope, ocean currents, precipitation, sea-surface temperature, geomorphic type (atoll vs island) and human population status (unpopulated vs populated). Generalized linear models (GLMs) revealed that geomorphic type, bathymetric slope, reef area and population status were the primary drivers of spatial variations in the IME among Pacific island- and atoll-reef ecosystems, together explaining 78% of the variation observed (*n*=28, only locations with significant increases in nearshore chlorophyll-*a* were modelled, *P* range=*<*0.0001–0.029, *r*^2^ range=0.59–0.99, see Supplementary Tables 3,4 and see Methods section).

**Figure 1:**
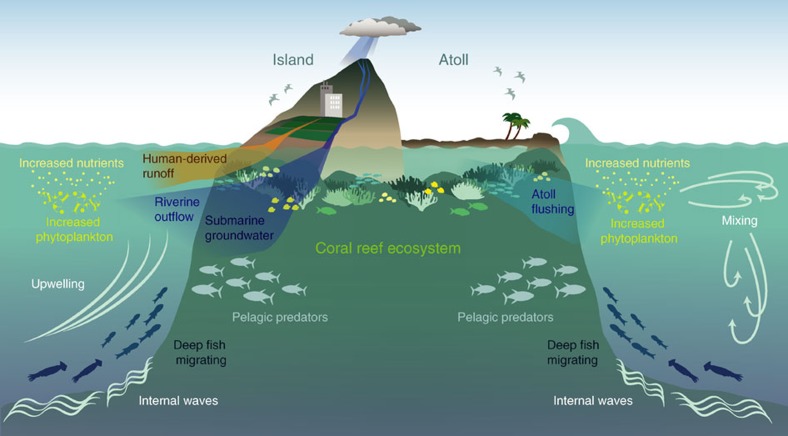
The Island Mass Effect. Localized increases in phytoplankton biomass near island- and atoll-reef ecosystems—Island Mass Effect—may be the result of several causative mechanisms that enhance nearshore nutrient concentrations, including coral reef ecosystem processes, such as nitrogen fixation or decomposition, and animal waste products, such as reef-associated fishes; current-bathymetric interactions that can drive vertical transport of water masses via upwelling, downstream mixing and eddies, and internal waves; island-associated inputs, such as submarine groundwater discharge and outflow from rivers, which can mobilize and transport sediment and other terrigenous material laden with nutrients; the flushing and associated outflow of lagoonal waters from atoll environments; human-derived runoff of agricultural production, urban development and wastewater input. Enhanced nearshore phytoplankton can influence food-web dynamics and elicit a biological response in higher trophic groups, for example: horizontal and vertical migration patterns in squids, fishes and other micronekton (collectively referred to as the ‘mesopelagic boundary layer community') that move nearshore at night to feed on increased food resources; inshore migration of pelagic predators, such as tuna, to feed on the island-associated micronekton community; greater reef fish biomass and increased cover of calcifying benthic organisms in coral reef ecosystems.

Variation in the IME across our study region was driven by differences in geomorphological make-up; nearshore chlorophyll-*a* enhancements were more pronounced at atolls than islands (34% of explained variation, Fig. 3a,b, Supplementary Table 4). Atolls, unlike islands, have partially enclosed interior lagoons often containing thriving ecosystems (Fig. 1). Wave- and tidal-driven flushing of these lagoons to surrounding waters may export nutrients fuelling enhanced nearshore phytoplankton biomass. Across our study system, the IME was most pronounced in the Northwestern Hawaiian Islands (Supplementary Table 1) at semi-enclosed atolls with naturally occurring channels to the open ocean. Large ocean swells generated from North Pacific storms and northeast trade-winds pump considerable amounts of water over the emergent barrier reef that then flow through the entire atoll system and eventually exit the channel^13^. Wave forcing is a highly efficient atoll flushing mechanism, advecting detritus and other sources of nutrients generated via coral reef ecosystem processes out of the atoll^14^. This rapid mobilization of material can exceed the assimilation ability of the benthic community^15^, thereby providing increased nutrients that drive nearshore phytoplankton biomass enhancement.

Along with geomorphic type, island- and atoll-reef ecosystems with more gradual sloping bathymetry exhibited a stronger IME (28% of explained variation, Fig. 3a, Supplementary Table 4). Bathymetric influences on ocean currents can force vertical transport of subsurface nutrient-rich waters that fuel nearshore productivity (Fig. 1). For example, vertical transport can be driven by current impingement that uplifts isotherms on the upstream side of an island^4^,^16^ or through turbulent mixing, lee eddy and wake effects on the downstream side^17^,^18^,^19^. Internal waves, generated from tidal currents interacting with underlying bathymetry, can also drive vertical perturbations in the background stratification that deliver cooler, nutrient-rich waters to the near-surface^20^ resulting in increased nearshore phytoplankton biomass^21^. The shoreward propagation of internal waves is directly related to bathymetric slope^22^; internal waves more readily reach shallower waters and fuel phytoplankton production where the underlying slope is more gradual. In contrast, internal waves are reflected offshore at steeper sloped locations. Across our study region, we found the IME to be particularly pronounced at locations within the Hawaiian Archipelago, a region characterized by islands and atolls with gradual sloping bathymetry (Supplementary Table 1) and highly active internal wave generation^23^.

Pacific island- and atoll-reef ecosystems with greater reef area exhibited increased nearshore chlorophyll-*a* enhancements and thus had a more pronounced IME (26% of explained variation, Fig. 3b, Supplementary Table 4). The mechanisms underlying this relationship include autochthonous nutrient sources in coral reef ecosystems such as nitrogen fixation, regeneration (either through decomposition of primary producers or from sediment deposition), and recycling from other biota^15^,^24^. Animal waste products, such as those derived from sea-bird guano^25^, reef-associated fishes^26^ and mobile marine invertebrates^27^ also enhance nutrient concentrations in coral reef ecosystems. The total reef-derived nutrients available to phytoplankton are likely variable, dependent upon biogeochemical processes within coral reef ecosystems that are influenced by physical factors such as water residence times and incoming light energy^15^,^24^. Nevertheless, total ecosystem processes presumably scales with total reef area, thereby driving increased phytoplankton biomass and an increased IME at larger island- and atoll-reef ecosystems.

A further 7% of overall variation was explained through an interaction between reef area and geomorphic type; chlorophyll-*a* enhancement was greater with increased reef area at islands vs atolls (Fig. 3a,b, Supplementary Table 4). Near island-reef ecosystems, phytoplankton biomass can be influenced by a variety of sources that increase ambient nutrient concentrations. For example, riverine outflow can export large amounts of nutrient-laden terrigenous material to the nearshore^28^, while submarine groundwater discharge can also drive considerable, albeit highly variable, increases in nearshore nutrient concentrations^29^. This significant interaction effect among drivers adds to a growing body of evidence that multiple and simultaneously changing biogeophysical drivers shape ecological communities in the marine realm.

The presence of local anthropogenic impacts also influenced variations in the IME; nearshore chlorophyll-*a* enhancements were greater at populated (*n*=7) than unpopulated (*n*=21) island- and atoll-reef ecosystems (12% of explained variation, Fig. 3a,b, Supplementary Table 4). Human activities can increase nearshore nutrient concentrations well beyond natural levels, artificially elevating planktonic production in coastal marine ecosystems^30^ (Fig. 1). This occurs through a variety of mechanisms including runoff from urban development and agricultural land use^28^. Wastewater effluent can also increase nearshore nutrient concentrations^31^, particularly in areas where treatment occurs on-site (for example, cesspools), a common waste disposal practice across the Pacific, including the heavily populated Main Hawaiian Islands^32^.

### Phytoplankton enhancement below the surface

Remotely sensed chlorophyll-*a* provides an estimate of phytoplankton biomass in the upper 10s of metres in the ocean^33^. However, phytoplankton biomass often increases deeper in the water column, reaching a subsurface maximum that can be far greater than that observed in surface waters^34^. Using ship-based surveys, we examined nearshore enhancements in phytoplankton biomass down through the upper water column (5–300 m, see Methods section). Across the 29 island- and atoll-reef ecosystems surveyed, depth-integrated chlorophyll-*a* levels increased towards shore at 73% (21 of 29) of locations (Fig. 4). This is clear evidence that the IME propagates well below surface waters and occurs over the full euphotic depth range.

While *in situ* surveys provided direct observations of nearshore phytoplankton enhancements in the upper water column, they also demonstrated clear spatiotemporal variability exists in the IME. Five locations surveyed (JAR, TUT, PHR, FFS and WAK; Fig. 4) exhibited temporal variation in the strength of nearshore subsurface phytoplankton enhancement. In contrast, three locations exhibited opposing spatial trends; phytoplankton biomass both increased and decreased towards shore within the same location during different survey years (data not shown). Temporal differences observed between surveys at individual island- and atoll-reef ecosystems likely reflect the variable nature of biogeophysical processes that drive increased nearshore phytoplankton biomass (Fig. 1). Current-topographic interactions and wave-driven lagoonal flushing, for example, exhibit spatiotemporal variability that can drive nutrient supply fluctuations resulting in a locally variable phytoplankton response^35^. Therefore, the extent of nearshore phytoplankton biomass enhancement may not be fully captured during our brief 1–3-day ship-based sampling efforts at a given island or atoll in a given year. Nevertheless, our *in situ* observations provide important verification that the observed surface phytoplankton biomass enhancements are indeed reflective of subsurface phytoplankton gradients near Pacific island- and atoll-reef ecosystems.

### Total increase in marine food resources

**Figure 2:**
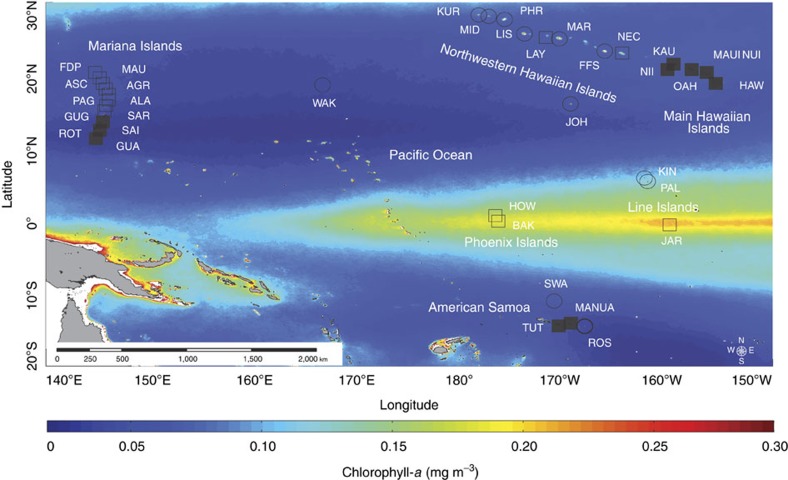
Map of the Pacific highlighting the coral reef islands and atolls used in this study. Long-term mean (10 year) chlorophyll-*a* with coral reef islands (squares) and atolls (circles) that either have local human populations (filled) or are unpopulated (open). Please see Supplementary Table 1 for study location name designations.

**Figure 3:**
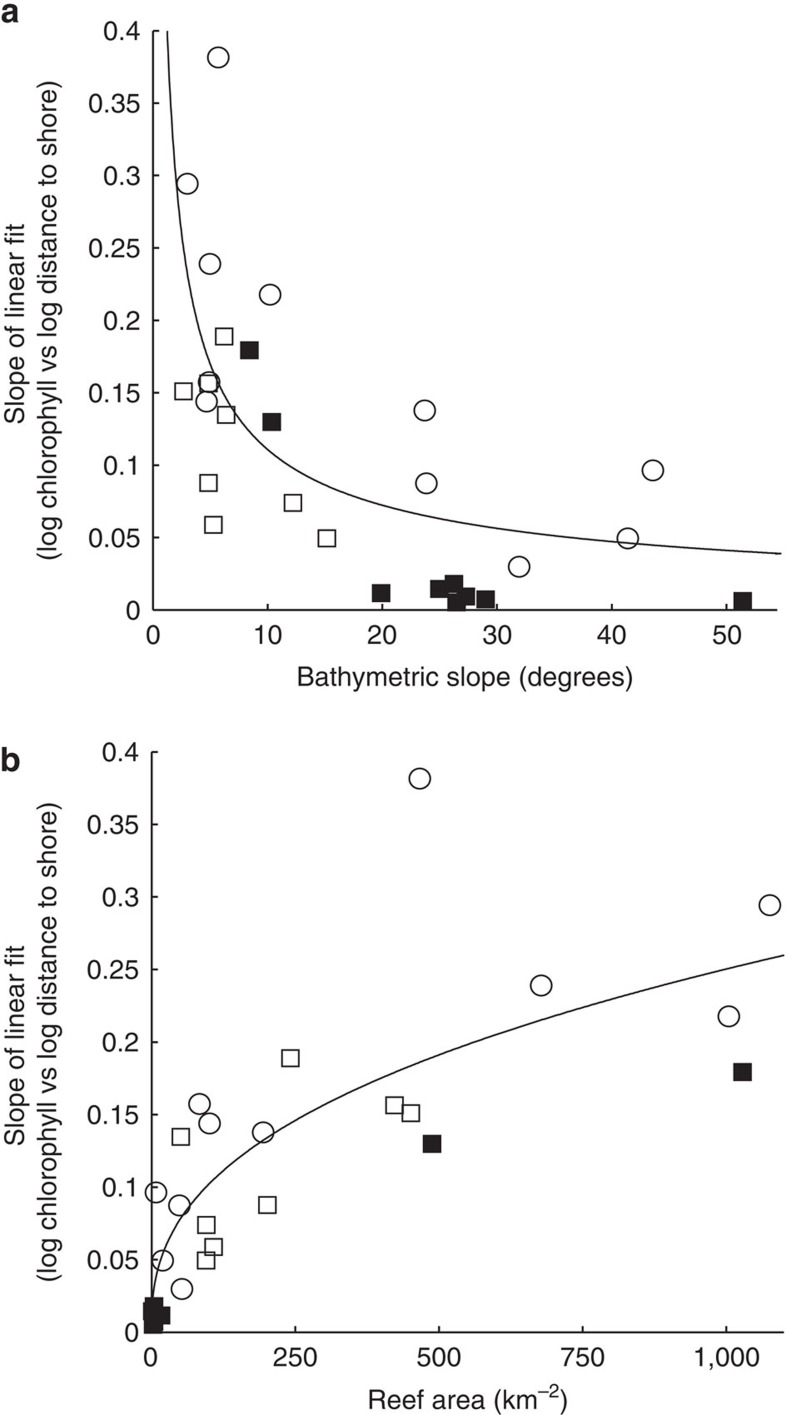
Biogeophysical drivers of variations in Island Mass Effect strength. Relationship between study locations that had significant phytoplankton biomass enhancement (*y*-axis; increasing *y* represents a greater rate of increase in chlorophyll-*a* towards shore) and significant drivers identified from model results; bathymetric slope (**a**) and reef area (**b**) with geomorphic type (atolls vs islands, represented as circles vs squares, respectively) and population status (populated vs unpopulated, represented as filled vs open icons, respectively). Geomorphic type and population status are identified the same as in Fig. 2. Nonlinear regressions (*P*<0.05) with *r*^2^ values of 0.45 (**a**) and 0.68 (**b**).

**Figure 4:**
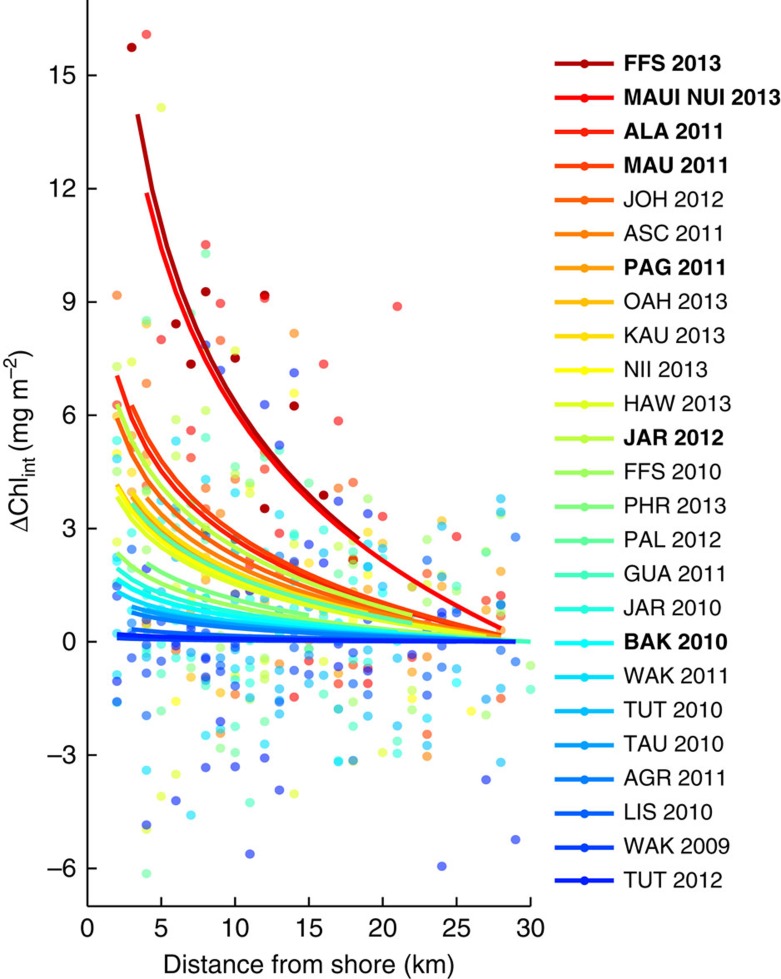
Phytoplankton enhancements below the surface. Relationship between changes in depth-integrated chlorophyll-*a* (ΔChl_int_) and distance from shore for 25 *in situ* surveys across 21 Pacific coral reef islands and atolls. Nonlinear regression fits are colour-coded based on the rate of increase in ΔChl_int_; red (blue) fits have a stronger (weaker) rate of increase in ΔChl_int_ towards shore across all surveys. Location and survey year are shown (right). Bold indicates significant relationships (*P*<0.05). All information is centred to have a value of zero Chl_int_ at 30 km, the spatial extent of satellite observations presented herein. Please see Fig. 2 for island and atoll geographic locations.

**Figure 5:**
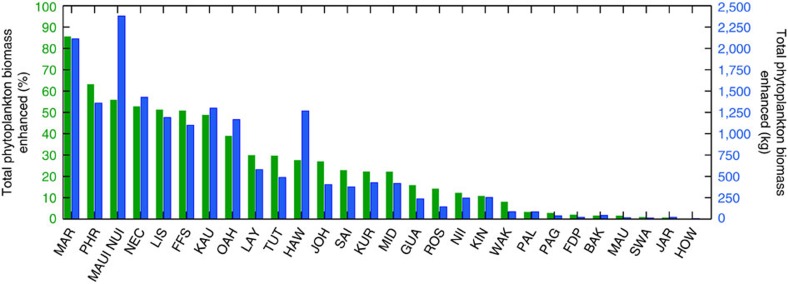
Total phytoplankton biomass enhanced near Pacific island- and atoll-reef ecosystems. Bars represent the total increase in long-term phytoplankton biomass at each location over offshore, oceanic phytoplankton biomass. Values are shown in percentage (green bars; left *y*-axis) and kg (blue bars; right *y*-axis), oriented in decreasing percent biomass from left to right. All locations had significant linear fits (*P*<0.05).

We examined the ecological implications of the IME by quantifying the total increase in standing stock of phytoplankton, and thus the increase in basal resources available to higher trophic levels, driven by the presence of each island- and atoll-reef ecosystem. The IME resulted in a long-term averaged (10 years) combined increase in nearshore phytoplankton biomass of 703.6% across our study locations (*n*=28; see Methods section). This represented a total increase of 17.21 metric tonnes over the background oceanic standing stock of phytoplankton. On a per island basis, we saw a range of 0.2–85.6% increase in total phytoplankton biomass (Fig. 5). The Hawaiian Archipelago harboured the top nine reef ecosystems with the greatest long-term increases in total phytoplankton biomass relative to background oceanic conditions (range; 29.9–85.6%). Other locations exhibited more modest enhancements in phytoplankton biomass. The equatorial islands of Howland, Baker and Jarvis, for example, showed a combined long-term enhancement in phytoplankton biomass of only 2.6% over background oceanic conditions (Fig. 5). This result was unexpected considering *in situ* observations of strong localized upwelling^16^, high cover of reef-building organisms^5^ and high planktivorous and predatory fish biomass^12^ at these locations. However, these islands are uniquely situated at the western edge of the equatorial cold tongue; a geographic area in the Pacific that experiences consistent trade wind-driven equatorial upwelling and high chlorophyll-*a* concentrations (Fig. 2). Therefore, a more muted biological response associated with the IME was observed at these island-reef ecosystems owing to the already elevated background phytoplankton levels.

## Discussion

The consequences of increased phytoplankton biomass span multiple trophic groups within coral reef island and atoll marine food-webs. Calcium carbonate-forming benthic organisms, namely hard (scleractinian) corals and crustose coralline algae, show increased abundance across the central and western Pacific Ocean when exposed to increased phytoplankton biomass^5^. Similarly, the biomass of planktivorous and piscivorous fishes and baseline estimates of Pacific reef sharks are far greater at locations with greater mean phytoplankton biomass^6^,^36^. In addition, a distinct community of squids, fishes and other deep-water associated micronekton are found in high densities near island-reef ecosystems relative to offshore waters^37^. This community—the mesopelagic boundary layer community—exhibits strong diel migration in association with subsurface island topography, transiting long distances (>5 km) towards shore at night and peaking in abundance and density in waters with increased phytoplankton biomass^38^. Pelagic predators, such as dolphins^39^ and tuna^40^ cue in on the shoreward migration of organisms, exploiting the island-associated micronekton community as a food resource (Fig. 1). Moreover, inter-island migratory patterns of marine apex predators, such as tiger sharks (*Galeocerdo cuvier*), appear to be driven by variations in phytoplankton biomass, presumably owing to net energetic gain associated with bottom-up driven increases in prey abundance^41^.

Phytoplankton biomass and the underlying drivers that enhance it may not always act to bolster ecological communities. For example, sewage pollution can increase nutrient levels that drive entire regime shifts in marine ecosystems^42^ while the associated phytoplankton enhancement can drive coastal eutrophication and toxic algal blooms, resulting in mass mortalities of fishes, marine mammals and seabirds^28^. In addition, cold ocean temperatures in chronic upwelling environments may limit coral growth and suppress reef-building processes that are important for coral reef persistence^43^. Similarly, upwelled nutrients can significantly exceed background concentrations and enhance fleshy (non reef-building) algal growth^20^, potentially disrupting benthic competitive interactions and reshaping coral reef communities. Because ecological communities can exhibit threshold-type responses, or tipping points, to variations in human^44^ and natural physical drivers^45^, similar relationships potentially exist between island- and atoll-reef ecosystems and the processes that enhance nearshore phytoplankton biomass. Future research is needed, however, to properly identify and better understand the existence of non-linearities in marine ecosystems associated with the IME.

Global shifts in biogeochemical cycling and ocean mixing associated with climate change are projected to decrease nutrient availability and primary production in the tropics and subtropics^46^, impacting fisheries and in turn compromising human food supplies^47^. However, state-of-the art climate models used in these projections do not resolve complex biophysical interactions, such as the IME, that occur at the island-reef ecosystem scale. The projected strengthening of the Pacific equatorial undercurrent^48^, for example, may increase vertical transport of nutrient-rich waters that drive phytoplankton biomass enhancements near equatorial island- and atoll-reef ecosystems despite projected regional declines in marine primary production. The number of island- and atoll-reef ecosystems that stand to benefit from future equatorial undercurrent strengthening is but a fraction of reef ecosystems in the Pacific. Nevertheless, these systems represent potentially important refugia for coral reef ecosystems and the food-webs they support in a rapidly changing climate. Biogeophysical drivers of the IME may also serve to bolster coral reef ecosystem resiliency to future thermal stress events and associated coral bleaching. The delivery of particle-laden deep ocean waters via upwelling and internal waves, for example, could provide important energetic subsidies^49^ and a thermal reprieve^50^ for corals during prolonged periods of anomalously warm temperatures.

Our basin-scale investigation of the IME demonstrates that nearshore phytoplankton enhancement is a long-term, near-ubiquitous feature among Pacific coral reef islands and atolls. Moreover, we found the magnitude of nearshore phytoplankton enhancement differed among island- and atoll-reef ecosystems owing to variations in key biogeophysical drivers, namely geomorphic type (atoll vs island), bathymetric slope, reef area and factors associated with the presence of local human populations. Individual coral reef island and atolls were capable of increasing the nearshore standing stock of phytoplankton biomass by up to 86% over background oceanic conditions, forming biological hotspots across an otherwise barren ocean landscape. Ecosystem services vital to human populations, such as fisheries production and coastal protection, are intrinsically linked to the nearshore phytoplankton enhancement associated with the IME. Such ecosystem-scale biophysical phenomenon must therefore be incorporated into future modelling efforts if we are to accurately predict the trajectory of marine ecosystems and the millions of people they support in this era of rapid change.

## Methods

### Quantifying chlorophyll-*a* enhancements

Spatial gradients in chlorophyll-*a* were quantified following Gove *et al.*^11^. In brief, long-term mean (July 2002 to June 2012) chlorophyll-*a* was calculated from 8-day, 0.0417° Moderate Resolution Imaging Spectroradiometer (MODIS) data. Eight sectors of ∼3.27 km width (0.0295°; ½ the diagonal of a satellite pixel) that were perpendicular to the 30 m isobath were quantified at each location (for example, Supplementary Fig.1). Sectors extended ∼3.27–6.54 km to ∼26.16–29.43 km (0.0295–0.0590° to 0.2360–0.2655°) offshore from the 30 m isobath. Data within ∼3.27 km of the 30 m isobath were removed to avoid optically shallow waters and errors induced by terrigenous input, re-suspended material or bottom substrate properties^51^ (Supplementary Fig. 2). Where island or atoll proximity resulted in sectors from different locations containing common data pixels, pixels were identified and removed before pixel averaging to avoid potential biases associated with the IME signal from one location influencing another's. However, when proximity resulted in a large proportion of pixels shared between locations (>50%), bathymetry was combined and sectors recalculated for the larger formed island-complex. Specifically, Molokai, Maui, Lanai and Kahoolawe were combined to form MAUI NUI; Saipan, Tinian and Agujian were combined to form SAI; Ofu, Olosega and Tau were combined to form MANUA (Fig. 2 and Supplementary Table 1).

We found the relationship between long-term chlorophyll-*a* and distance to shore was best described by a power function at all locations (for example, Supplementary Fig.1b). Given that a power function is equivalent to a linear fit on log–log transformed data, seamless transition can be made between nonlinear and linear fits and associated analyses. To test for differences in the relationship between long-term chlorophyll-*a* and distance to shore between locations, an analysis of covariance was performed on log–log transformed data. Two tests were performed; one test that included all islands and atolls (*n*=35) and another that only included locations that showed a significant increase in chlorophyll-*a* with decrease distance to shore (*n*=28; *P*<0.05).

### Biogeophysical drivers

We incorporated a series of biogeophysical parameters that are potential drivers of increased phytoplankton biomass near oceanic island- and atoll-reef ecosystems. The following were quantified for each location: latitude, geomorphic type (atoll vs island), reef area, land area, bathymetric slope, elevation, human population status (unpopulated vs populated) and the long-term mean and standard deviation for: sea-surface temperature, precipitation and ocean currents (Supplementary Table 2).

Latitude (°) represented the centre point of each location. Geomorphic type was either ‘atoll' or ‘island'. Reef area (km^2^) was calculated from the shore-line to the 30-m isobath and land area (km^2^) was calculated for all emergent land. Bathymetric slope (°) was derived from bathymetric grids in ArcGIS v10.1 using the Spatial Analyst ‘slope' function, calculated between 30–300 m depth and then averaged across the entire location. A detailed description of these factors (latitude, geomorphic type, reef area and land area) can be obtained in Gove *et al.*^11^.

Elevation (m) was obtained from a variety of sources, including the U.S. Central Intelligence Agency (https://www.cia.gov), NOAA's Coral Reef Information System (http://www.coris.noaa.gov) and the U.S. Fish and Wildlife Service (www.fws.gov). Population status was either ‘unpopulated' or ‘populated' following Williams *et al.*.^12^ Locations were considered populated with a human habitation of >160 people.

Island- and atoll-scale SST (°C) was obtained following Gove *et al.*^11^. In brief, SST was quantified using 0.0439°, 7-day information from the Pathfinder v5.0 data set (http://pathfinder.nodc.noaa.gov). Data were excluded if deemed of poor quality (quality value<4 ref. 52) or if individual pixels were masked as land. Island- and atoll-specific SST data were produced by spatially averaging the individual pixels that were intersected by or contained within the 30 m isobath for each location.

Precipitation data was obtained from the Global Precipitation Climatology Project v2.2 (http://www.esrl.noaa.gov); a global, 2.5° spatial resolution, monthly data set that merges remotely sensed (microwave and infrared) and surface rain gauge observations.

Ocean current data were obtained from NOAA's OSCAR (http://www.oscar.noaa.gov/); a global, 1° spatial resolution, monthly ocean current data set derived from satellite altimetry (sea-level) and scatterometer (wind). The magnitude of current was calculated from the zonal (u) and meridional (v) components of flow for each time step. Grid cells for precipitation and ocean currents were chosen based on the centre point of each island or atoll.

The long-term mean and the standard deviation were calculated for SST, precipitation and ocean currents for each location over the 10-year time period concurrent with the long-term chlorophyll-*a* values (July 2002 to June 2012). Where locations were combined to form a larger island-complex (that is, MAUI NUI, MANUA and SAI), time series data among islands were averaged for each time step before long-term mean and standard deviation calculations while remaining biogeophysical metrics were summed (land area, reef area), averaged (slope, latitude) or the maximum value was obtained (elevation).

Underlying bathymetry data for all locations were provided by the Pacific Islands Benthic Habitat Mapping Center (PIBHMC), Hawaii Mapping Research Group (HMRG), National Geophysical Data Center (NGDC) and satellite-derived global topography.

### Statistical analysis and model selection

Our research hypothesis was that the IME varied with island type (atoll vs island), reef area, land area, bathymetric slope, elevation, population status (populated vs unpopulated), SST (mean and standard deviation), precipitation (mean and standard deviation) and current speed (mean and standard deviation). We used slope (‘b') from the regression output of log–log transformed data (chlorophyll-*a* vs distance to shore) to represent the IME (that is, the response variable), enabling a standardized comparison in chlorophyll-*a* gradients among study locations. This hypothesis was tested by initially fitting a GLM. While also assuming linear relationships among response and predictor variables (that is, like traditional linear regression), GLMs relax the requirement of a normal error distribution, ‘generalizing' the model to other distributions (within the exponential family) by relating the response and predictor variables through a ‘link' function. Only locations with significant negative relationships (that is, increased chlorophyll-*a* with decreased distance to shore) were used in the model (*n*=28; *P*<0.05). Initial examination of response vs predictor(s) appeared to show a combination of linear and non-linear relationships were possible. A GLM was used as the starting model and the assumption of linearity (that is, use of GLM) was tested (as were all other assumptions). An error distribution of gamma was assumed (requiring a positive transformation of slope values before model input) and a log link was used.

Collinearity among predictors was tested both by calculating Pearson's correlation values and variance inflation factors (VIF, for multicollinearity; for example^53^ via car package^54^). Predictors with the highest VIF value were inspected with respect to their linear correlation with other predictors (via Pearson's correlation), as well as the mechanistic underpinnings of the research hypothesis. Following removal of the predictor representing the most concern (for example, highest collinearity), the model was refit and remaining predictors were assessed until all VIFs were <3 ref. 53. Following this iterative process, elevation, latitude, SST (mean and standard deviation) and the standard deviations of current speed and precipitation were removed. Remaining predictors were geomorphic type (atoll vs island), reef area, land area, bathymetric slope, population status (populated vs unpopulated), mean precipitation and mean current speed. Model variants representing all possible combinations of these predictors (main effects, see interactions below) were tested and the models were ranked according to Akaike Information Criteria corrected (AICc) for small sample size. Two candidate models were chosen based on ΔAICc≤2 (Supplementary Table 3). On the basis of these results (Supplementary Table 3), models representing all possible combinations of main effects and two-way interactions were tested with geomorphic type, reef area, bathymetric slope, population status and mean current speed as predictors (MuMIn package^55^). The interaction between geomorphic type and population status was removed as no atolls were populated. Two models were chosen based on ΔAICc≤2 (Supplementary Table 3). The highest AICc weight occurred for the simpler model and further analysis indicated mean current speed did not significantly improve model explanatory power (based on analysis of deviance via *χ*^2^-test, *P*=0.219). In contrast, the interaction term between geomorphic type (island) and reef area was significant (based on analysis of deviance via *χ*^2^- test, *P*=0.001), increasing the overall explained deviance of the model from 78 to 85%. The resulting best-specified model was:

(1) abs(b)∼Geomorphic Type+Reef Area+Bathymetric Slope+Population Status+Reef Area:Geomorphic Type

Next, the assumption of independence of response estimates was tested by fitting a generalized linear mixed model (via the lme4 package^56^) and including region (see Supplementary Table 1) as a random effect on the intercept as:

(2) abs(b)∼Geomorphic Type+Reef Area+Bathymetric Slope+Population Status+Reef Area:Geomorphic Type+(1|Region)

There was no significant difference found when comparing the model with and without this random effect (based on analysis of deviance via *χ*^2^- test, *P*=0.5) and the fixed effects model was chosen (model 1).

The assumption of linearity of relationships among predictors and response was then tested by refitting model 1 as a Generalized Additive Model (GAM; mgcv package^57^) which included smoothing terms on the continuous variables of slope and reef area (no interaction terms possible):

(3) Geomorphic Type+s(Reef Area)+ s(Bathymetric Slope)+Population Status

There was a significant difference between the GLM (model 1) and GAM (model 3) with *P*=0.012 (analysis of deviance via *χ*^2^-test). The GLM is simpler and with a lower AICc (GLM AICc: −105 vs GAM AICc: −95) and thus the GLM (model 1) was chosen as our best-fit model.

The resulting model exhibited well-behaved uniform residuals with no significant outliers. Residuals deviated somewhat from an ideal normal distribution when inspected graphically but this deviation was not significant (that is, residual distribution did not differ significantly from normal; Shapiro–Wilks *P*=0.12). Models were refit with alternate link functions (for example, ‘inverse') but models fit with a log link function provided the most well-behaved residuals. The model was significant with *P*<0.0001 and explains 85% of the overall deviance. Predictor coefficients and significance are shown in Supplementary Table 4.

Chlorophyll enhancement increases (that is, slope b is more negative) at atolls (vs islands; Wald's test *P*<0.0001), decreases in bathymetric slope (Wald's test *P*=0.004) and at populated locations (vs unpopulated; Wald's test *P*=0.001). While reef area on its own was not significant (Wald's test *P*=0.27), there is a significant interaction term between reef area and geomorphic type that indicates that chlorophyll enhancement increases (b is more negative) with increased reef area at islands more than atolls (Wald's test *P*=0.002). In all cases, the magnitude of enhancement will be relative to the surrounding waters (vs absolute chlorophyll abundance).

The relative importance of each predictor in explaining the deviance was determined by hierarchical partitioning, which examines the effect of removing each predictor from models representing all possible orders of variables (Supplementary Table 4). In this way, the average independent contribution to explained deviance of each predictor is obtained^58^. Hierarchical partitioning was performed using the hier.part package in R^59^ (note that interaction terms are not included in this analysis) and indicated that relative importance of geomorphic type, bathymetric slope, reef area and population status in explaining the overall deviance of 34, 28, 26 and 12%, respectively. As above, these predictors explained 78% of the overall variability in the data, with the interaction term of reef area and geomorphic type increasing the explained deviance to 85%. All data manipulation and statistical analyses were performed using Matlab v2013b and R (R Core Team 2013 and related packages) unless otherwise specified.

### Total phytoplankton biomass

The total long-term mean (July 2002 to June 2012) in nearshore standing stock phytoplankton biomass enhanced by each island and atoll over background oceanic phytoplankton was calculated using remotely sensed observations. We first calculated the long-term mean in depth-integrated chlorophyll-*a* (ΣChl; mg m^−2^) by multiplying the long-term mean in chlorophyll-*a* (see above for details) by the long-term mean in the depth of light penetration for each satellite pixel. Depth of light penetration, or the depth at which the satellite can ‘see' into the water column, was calculated by taking the reciprocal of the long-term mean in 8 day, 0.0417° MODIS k490 ref. 33. All long-term mean ΣChl values within each sector (see Supplementary Fig.1) were then averaged for each island and atoll. The relationship between ΣChl and distance to shore was then quantified by applying a linear fit on log–log transformed data. Only locations with significant (*P*<0.05) negative relationships (that is, increased ΣChl with decreased distance to shore) were used (*n*=28; same locations used in modelling efforts). Using the furthest sector from each location (sector 8, for example see Supplementary Fig.1) to represent offshore, oceanic conditions in phytoplankton, we calculated the change (Δ) in ΣChl with each subsequent, more proximate sector. We then multiplied ΔΣChl for each sector by the respective sector area (m^2^; the change in latitude was accounted for in longitude to distance conversions for all locations) to calculate the phytoplankton enhanced (kg and percentage) for each sector. Summing over all sectors (1–7) enabled a quantitative estimate of the long-term phytoplankton biomass enhanced by the presence of each island and atoll in our study.

### Ship-based phytoplankton

Vertical chlorophyll-*a* profiles were obtained using a profiling fluorometer during 37 ship-based surveys of 29 individual islands and atolls in our study region. Surveys consisted of horizontal transects, starting ∼2–4 km from shore and extending 20–30 km offshore in one or more cardinal directions over 1–3 days. Depth-integrated *in situ* chlorophyll-*a* (mg m^−2^) was calculated over the upper water column (5–300 m depth). Nonlinear least squares regression fits were applied over the spatial distance covered within each survey.

## Additional information

**How to cite this article:** Gove, J. M. *et al.* Near-island biological hotspots in barren ocean basins. *Nat. Commun.* 7:10581 doi: 10.1038/ncomms10581 (2016).

## Supplementary Material

Supplementary Information Supplementary Figures 1-2 and Supplementary Tables 1-4
