# Supplementary material for: Near-island biological hotspots in barren ocean basins
Source: Nat Commun. 2016 Feb 16;7:10581. doi: 10.1038/ncomms10581 (PMC4757766; doi:10.1038/ncomms10581)
Supplement: Supplementary Information — Supplementary Figures 1-2 and Supplementary Tables 1-4 [file ncomms10581-s1.pdf]

Supplementary Information

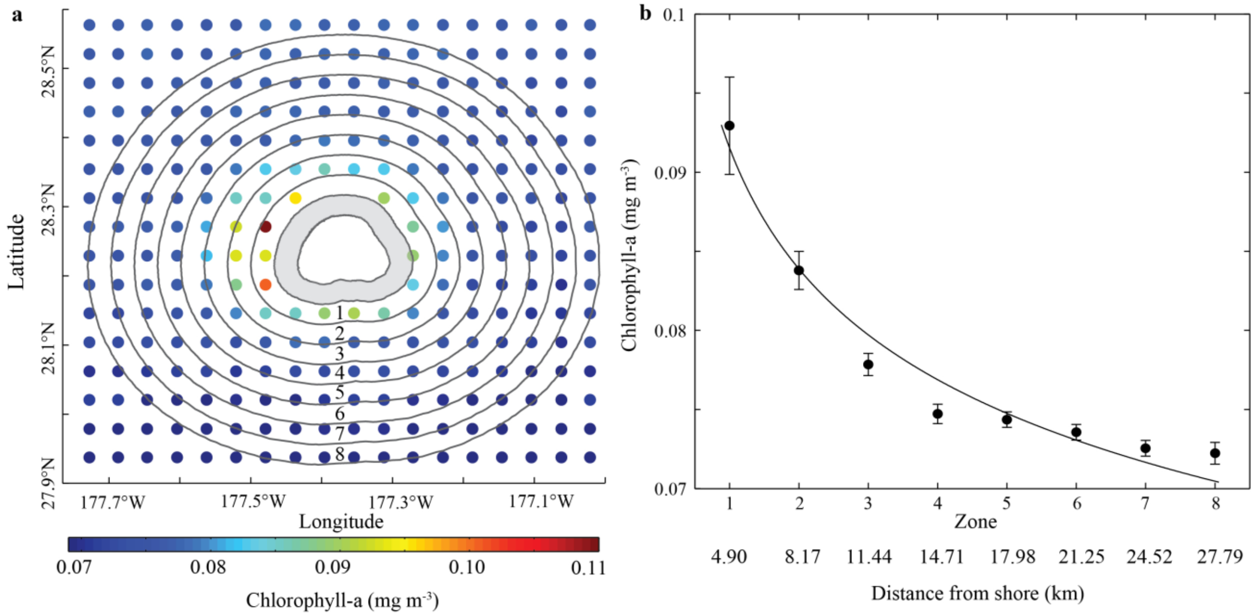

Supplementary Figure 1| Quantifying nearshore phytoplankton enhancement associated with the Island Mass Effect. **a**, Example location (Midway Atoll) of long-term (10 year) mean chlorophyll-*a* highlighting the spatially expanding, non-overlapping data sectors (black lines), numbered 1 – 8. Gray area represents data removal filter applied to exclude biased information associated with optically shallow waters (see Supplementary Figure 2). **b**, Long-term mean chlorophyll-*a* values (± standard error) calculated by averaging all pixels within each sector shown in **a**. Significant (P <0.05) nonlinear least squares regression line shown with an $R^2 = 0.95$. The numbers on the *x*-axis are associated with each sector shown in **a**. Distance from shore (km) based on the center point of each sector is also shown for ease of interpretation.

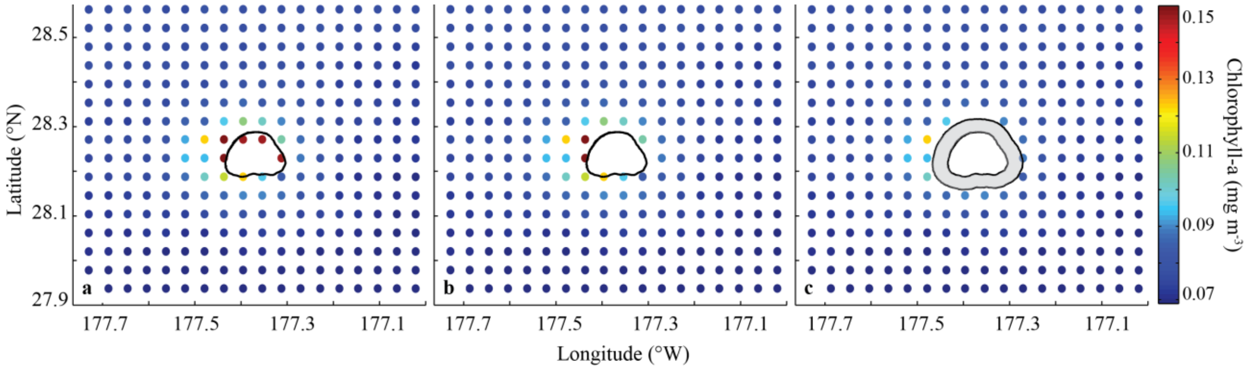

Supplementary Figure 2| Quality control of remotely sensed chlorophyll-*a* data near island- and atoll-reef ecosystems. Long-term averaged (10 year) chlorophyll at Midway Atoll, located in the Northwestern Hawaiian Islands (Fig. 2a). **a**, 30-m contour (black line) with unfiltered, contaminated information associated with shallow-water bottom reflectance. **b**, Data filtered using the 30-m bathymetric contour (black line), although contaminated information still remains as a result of bottom reflectance. **c**, Fully cleaned data set using an additional data removal filter (gray area) that is everywhere perpendicular to the 30-m contour, removing all contaminated data associated with bottom reflectance

Supplementary Table 1| Coral reef islands and atolls used to study the Island Mass Effect. Locations are oriented based on the slope of a linear fit of long-term chlorophyll-*a* and distance to shore (i.e. linear fit on log-log transformed chlorophyll-*a* and distance to shore), from strongest to weakest. *Location Name* is the name of the island(s) or atoll(s), *Location Code* is the three-letter code used in Fig. 2a. Region is the geopolitical region, where NWHI = Northwestern Hawaiian Islands, MHI = Main Hawaiian Islands, AMSAM = American Samoa, PRIA = Pacific Remote Island Areas, and MARIANA = Mariana Archipelago. *Geomorphic Type* (*Atoll or Island*) is based on primary geomorphological make up. *Lat* (latitude) and *Lon* (longitude) are in decimal degrees north and east, respectively, based on the center point of each location. *Reef Area* (km^2) is calculated from 0 – 30 m. *Bathymetric Slope* (°) represents the average calculated from 30 – 300 m. *Population Status* is either Unpopulated *(U) or* Populated *(P)*, based on a human population of 160 people. Location Codes in bold represent islands and atolls that had significant ($P < 0.05$) fits and were used in the generalized linear models.

Location Name	Location Code	Region	Geomorphic Type	Lat	Lon	Reef Area	Bathymetric Slope	Population Status
Pearl & Hermes Reef	**PHR**	NWHI	Atoll	27.86	-175.85	467.27	5.73	U
Maro Reef	**MAR**	NWHI	Atoll	25.41	-170.58	1075.44	3.03	U
French Frigate Shoals	**FFS**	NWHI	Atoll	23.79	-166.21	677.96	4.96	U
Lisianski	**LIS**	NWHI	Atoll	26.01	-173.95	1004.27	10.21	U
Kauai	**KAU**	MHI	Island	22.09	-159.57	241.70	6.22	P
Necker	**NEC**	NWHI	Island	23.58	-164.70	1028.32	8.4	U
Kure	**KUR**	NWHI	Atoll	28.42	-178.33	83.15	4.87	U
Oahu	**OAH**	MHI	Island	21.49	-158.00	422.72	4.84	P
Maui, Lanai, Molokai, Lanai, Kahoolawe	**MAUI NUI**	MHI	Island	20.83	-156.75	450.84	2.68	P
Midway	**MID**	NWHI	Atoll	28.23	-177.38	101.52	4.7	U
Johnston	**JOH**	PRIA	Atoll	16.74	-169.52	194.01	23.73	U
Tutuila	**TUT**	AMSAM	Island	-14.30	-170.70	50.89	6.39	P
Laysan	**LAY**	NWHI	Island	25.78	-171.73	488.13	10.36	U
Rose	**ROS**	AMSAM	Atoll	-14.55	-168.16	7.80	43.61	U
Hawaii	**HAW**	MHI	Island	19.53	-155.42	201.67	4.84	P
Kingman	**KIN**	PRIA	Atoll	6.40	-162.38	47.63	23.88	U
Saipan, Tinian, Aguijan	**SAI**	MARIANA	Island	15.01	145.65	95.15	12.20	P
Niihau	**NII**	NWHI	Island	21.90	-160.15	108.06	5.27	P
Wake	**WAK**	PRIA	Atoll	19.30	166.62	19.18	41.4	U
Guam	**GUA**	MARIANA	Island	13.46	144.79	94.85	15.16	P
Palmyra	**PAL**	PRIA	Atoll	5.88	-162.09	52.50	31.91	U
Baker	**BAK**	PRIA	Island	0.20	-176.48	4.43	26.24	U
Farallon de Pajaros	**FDP**	MARIANA	Island	20.55	144.89	1.38	24.98	U
Pagan	**PAG**	MARIANA	Island	18.11	145.76	16.29	19.89	U
Maug	**MAU**	MARIANA	Island	20.02	145.22	3.17	27.28	U
Ofu, Olosega, Tau	MANUA	AMSAM	Island	-14.21	-169.56	22.42	20.53	P
Jarvis	JAR	PRIA	Island	-0.37	-160.00	4.32	29	U
Swains	SWA	AMSAM	Island	-11.06	-171.08	2.82	51.41	U
Howland	HOW	PRIA	Island	0.80	-176.62	2.57	26.48	U
Guguan	GUG	MARIANA	Island	17.31	145.84	2.00	21.46	U
Sarigan	SAR	MARIANA	Island	16.71	145.78	2.00	16.74	U
Asuncion	ASC	MARIANA	Island	19.69	145.40	2.54	16.32	U
Alamagan	ALA	MARIANA	Island	17.60	145.83	4.28	23.65	U
Rota	ROT	MARIANA	Island	14.16	145.21	16.03	12.34	P
Agrihan	AGR	MARIANA	Island	18.76	145.66	9.50	23.03	U

Supplementary Table 2| Biogeophysical predictor variables investigated as proximate drivers of the Island Mass Effect. Summary of information for predictor variables that were calculated for each individual island- and atoll-reef ecosystem across our study system. Predictors that were highly collinear were removed during model selection efforts and are indicated as *NO* under *Included after estimation of collinearity*.

**Predictor**	**Units**	**Relevant Information**	**Source**	**Included after estimation of collinearity**
Latitude	Degrees	Center point of each location	Gove et al., 2013	NO
Land Area	km^{-2}	Average area of all emergent land	Gove et al., 2013	YES
Reef Area	km^{-2}	Average area from 0 – 30 m	Gove et al., 2013	YES
Bathymetric Slope	Degrees	Calculated between 30 – 300 m and averaged over the entire location	See methods	YES
Ocean Currents	$m\ s^{-1}$	1° spatial resolution, monthly data	NOAA's OSCAR (Ocean Surface Current Analysis – Real time)	YES
Precipitation	$mm\ d^{-1}$	2.5° spatial resolution, monthly data	NOAA's Global Precipitation Climatology Project v2.2	YES
SST	°C	Island and atoll specific data set derived from 4km, weekly data	Gove et al., 2013	NO
Geomorphic Type	Atoll/Island	Based on primary geomorphological make up	Gove et al., 2013	YES
Population Status	Unpopulated/ Populated	Locations were considered 'populated' with a human population of >160 people.	Williams et al., 2011	YES

Supplementary Table 3| Summary of model results. Comparisons of "best-fit" models selected among models representing all possible predictor combinations (see Methods for selection of predictors) based on AICc. Top candidate models were selected based a ΔAICc of <= 2 first among main effects models and then, based on these results, for models representing all remaining main effects and their two-way interactions. Best-fit models were subsequently assessed for their ability to meet model assumptions as well as the possible influence of correlation in space among response estimates (please see Methods for more information).

**Predictors**	**Number of Predictors**	**Log Likelihood**	**AICc**	**ΔAICc**
Main Effects				
Geomorphic Type (Atoll) + Bathymetric Slope + Reef Area + Population Status (Populated)	4	56.162	-96.323	0
Geomorphic Type (Atoll) + Bathymetric Slope + Reef Area + Population Status (Populated) + Mean Current	5	57.814	-96.028	0.295
Main Effects + Interactions				
Geomorphic Type (Atoll) + Bathymetric Slope + Reef Area + Population Status (Populated) + Reef Area:Geomorphic Type (Island)	5	62.146	-104.7	0
Geomorphic Type (Atoll) + Bathymetric Slope + Reef Area + Population Status (Populated) + Mean Current + Reef Area:Geomorphic Type (Island)	6	63.221	-102.9	1.83

Supplementary Table 4| Parameter estimates, significance and explanatory power of predictors from the best-specified model (eq. 1; Methods). The relative importance of each predictor in explaining overall deviance was determined via hierarchical partitioning (please see Methods for more information).

Predictors	Coefficient Estimate	P value	Deviance Explained (%)
(Intercept)	0.21	< 0.0001	NA
Geomorphic Type (Atoll)	0.13	< 0.0001	34
Bathymetric Slope	0.97	< 0.0001	28
Reef Area	1.00	0.27	26
Population Status (Populated)	3.00	<0.01	12
Reef Area:Geomorphic Type (Island)	1.00	<0.01	NA
